# An observational study of the marketing practice of e-cigarette specialty stores in two large cities in China: Is there potential to normalize the use of e-cigarettes?

**DOI:** 10.18332/tid/191840

**Published:** 2024-09-14

**Authors:** Hui Deng, Ling Fang, Lingyun Zhang, Sisi Wen, Shuai Zhang, Fan Wang, Pinpin Zheng

**Affiliations:** 1 Department of Preventive Medicine and Health Education, School of Public Health, Fudan University, Shanghai, People's Republic of China; 2 Health Communication Institute, Fudan University, Shanghai, People's Republic of China; 3 Key Lab of Public Health Safety of Ministry of Education, Fudan University, Shanghai, People's Republic of China; 4 Tobacco-Free Kids Action Fund Beijing Representative Office, Beijing, People's Republic of China; 5 The Research Center for Food and Drug Law, School of Law-based Government, China University of Political Science and Law, Beijing, People's Republic of China; 6 Fudan Development Institute, Fudan University, Shanghai, People's Republic of China

**Keywords:** social marketing, e-cigarette, advertising and promotion

## Abstract

**INTRODUCTION:**

Supervision measures in China have designated offline retail as the only legal channel for the sale and advertising of e-cigarettes. Specialty stores, exclusively selling vaping devices and e-liquids, are professionally designed to showcase company images and provide the best examples of e-cigarette marketing strategies. The goal was to analyze the retail marketing practice of e-cigarette specialty stores and provide a scientific reference for future e-cigarette point-of-sale regulation.

**METHODS:**

On-site observations were conducted in specialty stores among the popular business districts of Chengdu and Shanghai, China, from January to May 2021. ‘Dianping’, known as ‘Chinese Yelp’, was used to identify 8 business districts in Shanghai and 5 in Chengdu as observation sites. Two trained observers visited each store in the identified business districts. The data were collected with a checklist, which consisted of 5 sections with 37 items, including basic information, marketing practice, age restriction and health warnings.

**RESULTS:**

In total, 161 e-cigarette specialty stores, including 82 specialty stores in Shanghai and 79 in Chengdu, were identified. Of these stores, 156 were single-brand retailers and 5 were multi-brand retailers. Each store displayed e-cigarette products, which were visible from outside the store. The most common e-cigarette products were rechargeable kits and nicotine-containing e-liquids, which were available at all specialty stores. Frequent forms of promotion were free e-liquid samples (100%) and slogans (57.8%). Signage stating prohibition of minor use and purchase was presented at 141 (87.6%) specialty stores. Relatively few specialty stores (31.7%) displayed health warnings.

**CONCLUSIONS:**

E-cigarette specialty stores featured highly visible product displays, varied product selections, abundant marketing materials, free trial services, absent entry restrictions for minors, and a lack of health warnings. Policymakers should move to reduce youth exposure to e-cigarette products and marketing in the retail environment by strengthening regulations on product display and marketing.

## INTRODUCTION

Awareness and use of e-cigarettes among adults and adolescents in China have increased in recent years. The prevalence of past 30-day e-cigarette use among Chinese adults increased from 1.3% in 2015–2016 to 1.6% in 2018–2019, which resulted in an estimated 16.9 million adults using e-cigarettes between 2018 and 2019^1^. Although the prevalence of e-cigarette use in China is seemingly lower than that in other countries, such as the United States^2^, the estimated population using e-cigarettes is considerable due to the large population.

The significant user base can be attributed to the growth of the e-cigarette industry. The data show that the size of the domestic retail market for e-cigarettes was approximately 19.7 billion RMB (US$3.09 billion) in 2021, with a year-to-year growth of 36%^3^. E-cigarette products are sold via three channels: online, traditional retailers (e.g. convenience stores), and specialty shops (that exclusively sell vaping devices and e-liquids). Chinese e-cigarette manufacturers used to focus on the online market, and e-cigarettes were initially promoted and sold online. Before 2018, e-cigarette online sales in China made up approximately 80% of the consumer market, while in-store sales constituted 14%^4^.

To safeguard minors from the allure of e-cigarettes, the Chinese government has implemented several regulations. In November 2019, the State Tobacco Monopoly Administration (STMA) and the State Administration for Market Regulation (SAMR) issued a notice calling for e-cigarette manufacturers and retailers to stop selling and advertising e-cigarettes online^5^. This notice effectively made offline retail the sole legal channel for e-cigarette sales and advertising, intensifying e-cigarette companies’ interest in the offline retailer market. In 2020, RELX Technology, a leading e-cigarette company in China, announced plans to invest 600 million RMB (US$87 million) over the subsequent three years, with the goal of establishing 10000 stores globally^6^. Following suit in 2021, other leading brands, such as SNOWPLUS, released similar expansion strategies. These investments were strategically allocated to bolster product distribution across various retail avenues, among which a significant portion was designated for starting specialty stores.

Since the abovementioned regulations lack supervision for offline retailers, e-cigarettes have become widely accessible through offline retailers across China. From 2018 to 2021, the number of offline e-cigarette retailers increased substantially. For example, RELX Technology, which opened its first specialty store in December 2018, expanded to greater than 24000 stores across 300 cities in China by the end of 2021^7^. According to the ‘Electronic Cigarette Industry Blue Book 2021’, there are approximately 190000 e-cigarette retail stores in China^3^. Of these, 47000 are one-brand specialty stores, and the number of multi-brand specialty stores ranges between 5000 and 7000.

Currently, offline retailers have become the most important marketing channel through which e-cigarette companies can communicate with current, former and potential users in China. Retailers use diverse marketing strategies, such as advertising and promotion, to expand markets, attract new users, promote continued use, and build brand loyalty^8^. However, even brief exposure to marketing can act as a catalyst for the initiation and subsequent use of e-cigarettes^9^. The existing advertising law in China prohibits advertising of tobacco products in media, public areas, public transport, and outdoor spaces^10^. Yet, the law does not clearly delineate what constitutes a public place. Many retail outlets still market tobacco products at the point-of-sale as if there are no relevant regulations applicable to them. Gaining insights into how e-cigarettes are marketed within retail environments is crucial for pinpointing the current situation, framing potential regulations, and guiding future research.

Many observational studies on e-cigarette retailers from the USA^11-16^, Canada^17^, Guatemala^18^, New Zealand^19^, and Australia^20^ have documented widespread availability and advertising. Many of these retailers use conventional promotion strategies, similar to those used for traditional tobacco products, including product displays and sales promotions^17,19^. Furthermore, e-cigarette stores build rapport with customers and create an atmosphere centered around vaping. This not only promotes interactions but also fosters a sense of community, to attract customers^21^. Despite the growing number of e-cigarette studies, to date, no studies on the marketing practices of e-cigarettes within the Chinese retail environment have been published. The frequency of exposure to in-store e-cigarette advertising correlates with an increased likelihood of e-cigarette use among youth^9^, underscoring the significance of recording the e-cigarette retail environment.

Given the limited research on e-cigarette retailers coupled with their pronounced expansion in China, the retail environment evidently needs to be closely investigated. Specialty stores, which exclusively sell vaping devices and e-liquids, are professionally designed for companies to show their image and provide the best examples of how e-cigarette companies market their product and brand. The goal was to analyze the retail marketing practice of e-cigarettes specialty stores and provide a scientific reference for future e-cigarette point-of-sale regulation.

## METHODS

This study was designed as an observational cross-sectional analysis to explore the marketing practices of e-cigarette specialty stores. The observational nature of the study allowed us to gather data on store characteristics and marketing strategies without intervening or altering the environment. The data were collected in two phases: 1) the identification of all e-cigarette specialty stores in the popular business areas in Shanghai and Chengdu; and 2) on-site observations at selected specialty stores.

### Sample

This study was conducted at e-cigarette specialty stores in Shanghai and Chengdu, which are the most developed cities with the largest number of e-cigarette stores^3^ in Eastern and Western China. To record detailed marketing information at e-cigarette specialty stores, we developed an observation checklist. This development process included: 1) reviewing the previous literature about e-cigarette retailers^11-17^, which allowed us to identify the marketing strategies used by e-cigarette retailers; and 2) observing 10 specialty stores in Shanghai, which helped us to identify additional strategies. The checklist was reviewed by the working group, refined and retested to generate consistent definitions. The final checklist consisted of 5 sections with 37 items, including basic information, product displays, promotion, age restrictions and health warnings (Supplementary file Table S1).

Given that e-cigarette stores were concentrated in popular and large business districts, we decided to conduct on-site observations in these popular business districts to quickly obtain observation samples. ‘Dianping’, which can also be referred to as ‘Chinese Yelp’, is a Chinese website that allows users to review and comment on businesses, restaurants and other service providers. Hence, we can find the most popular business districts in the city according to their rankings on ‘Dianping’. For inclusion in the study, specialty stores had to exclusively sell e-cigarette products and accessories, including one-brand and multi-brand specialty stores, and not sell other products, including cigarettes, cigars, or other tobacco.

### Procedure

Through ‘Dianping’ rankings, we identified 8 business districts in Shanghai and 5 in Chengdu for further observation. The observation areas in Shanghai covered the districts of Pudong New, Huangpu, Yangpu, Xuhui, Jing’an, Changning, and Minhang. The observation areas in Chengdu included the districts of Jinjiang, Chenghua, Qingyang, and Wuhou. On-site observations were conducted from January to May 2021. Two trained observers visited each shopping mall, covering every floor, and explored all the shops on every street within each identified business district. This comprehensive survey included all e-cigarette specialty stores, ensuring that our observations encompassed the entire scope of the retail landscape in these areas. Approximately 10 to 15 min were spent in each specialty store. Our observational visits took place from 9 a.m. to 10 p.m., encompassing both weekdays and weekends. During these visits, the observers conducted thorough walkthroughs, covering every aisle in each store, to gain an in-depth understanding of the overall situation. The owners of the shop were not informed in advance about the store visit to avoid any ‘preparation’. Consent from the retailer was not needed. Ultimately, we identified 82 specialty stores in Shanghai and 79 in Chengdu and completed observations in these 161 specialty stores (Supplementary file Table S2).

The data were gathered using a specifically designed checklist (Supplementary file Table S1) that covered various aspects: the type of specialty store (either single brand or multiple brands), the brand name, available flavors, product types (categorized as disposable or rechargeable kits), the availability of zero-nicotine e-liquids (yes or no), the presence of discounts (yes or no), marketing materials, and the existence of signage indicating a minimum age requirement or a no-sale-to-minors policy (yes or no). One observer was responsible for completing the questionnaire, and another observer conducted an on-the-spot review of the completed checklist to ensure the consistency of the observational content. The study protocol received approval from the Ethics Committee of School of Public Health, Fudan University (IRB#2021-04-0898).

### Statistical analysis

The store was used as the unit of analysis. Descriptive statistics were computed to characterize product types, promotions, product displays, marketing materials and other shop characteristics. Chi-squared test or Fisher’s exact test was used to compare these attributes between the two cities. SPSS V.20.0 was used for all the statistical analyses.

## RESULTS

### Basic information

**Figure 1:**
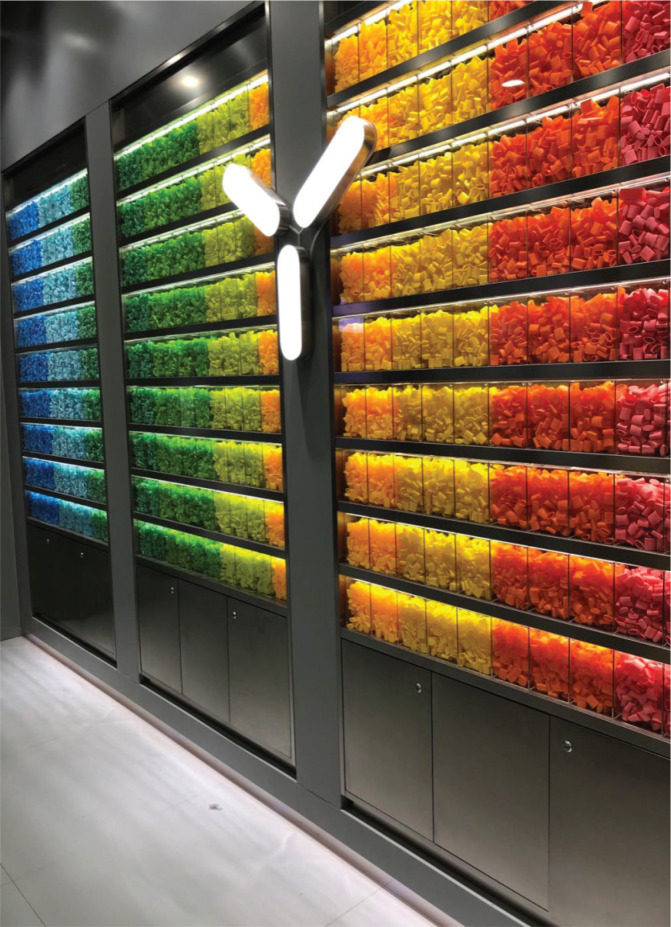
YOOZ specialty store featuring disposable plastic cover decorations on its background wall

Of the stores, 156 were single-brand retailers and 5 were multi-brand retailers (Table 1). There were no significant differences between single-brand and multi-brand stores in the two cities. Twenty-three distinct brands, including RELX, SNOWPLUS, and YOOZ, were available. Notably, approximately 80% of these specialty stores were <10 m^2^ in size. The majority of these shops were situated at either underground (37.8%) or the ground floor (46.0%). A noticeable characteristic of single-brand stores was the consistent and modern decoration style in different stores of the same brand. For example, YOOZ, a leading manufacturer in China, uses disposable plastic covers to decorate specialty store background walls, which create a distinctive brand identity that resonates with their target customers (Figure 1).

**Table 1:** Basic information of e-cigarette specialty stores (N=161)

*Basic information*	*Total n (%)*	*Shanghai n (%)*	*Chengdu n (%)*	*p*
**Specialty store type**				0.170
Single brand	156 (96.9)	79 (96.3)	77 (97.5)	
Multiple brand	5 (3.1)	3 (3.7)	2 (2.5)	
**Area** (m^2^)				0.049
0–10	124 (77.0)	69 (84.1)	55 (69.6)	
11–20	32 (19.9)	9 (11.0)	23 (29.2)	
>20	5 (3.1)	4 (4.9)	1 (1.3)	
**Location**				<0.001
Inside shopping malls	115 (71.4)	65 (79.3)	50 (63.3)	
Along the streets	41 (25.5)	12 (14.6)	29 (36.7)	
Around the metro station	5 (3.1)	5 (6.1)	0 (0)	
**Floor**				<0.001
Lower basement	26 (16.1)	8 (9.8)	18 (22.8)	
Basement	35 (21.7)	28 (34.1)	7 (8.9)	
Ground floor	74 (46.0)	25 (30.5)	49 (62.0)	
First floor	11 (6.8)	8 (9.8)	3 (3.8)	
Second floor or higher	15 (9.3)	13 (15.9)	2 (2.5)	

### Product display

All of the retailers displayed their e-cigarette products (Table 2), which were highly visible from outside the stores (n=161; 100%). Rechargeable kits, including e-liquid refills, were sold at all stores, and disposable e-cigarettes were sold at 54.0% of the stores. Disposable and rechargeable e-cigarettes were available in a wide variety of flavors, and the average number of e-liquid flavors offered in each store was 30.3 ± 1.4. E-liquid with nicotine was the most common available type, present in all 161 (100%) of the observed shops. Additionally, 18 specialty stores (11.2%) offered non-nicotine e-liquids. There were no significant differences in product characteristics in the two cities.

**Table 2:** Sale practices of e-cigarette specialty stores (N=161)

*Sale practices*	*Total n (%)*	*Shanghai n (%)*	*Chengdu n (%)*	*p*
**Products**	161 (100)	82 (100)	79 (100)	1
Disposable	87 (54.0)	40 (48.8)	47 (59.5)	0.228
Rechargeable kits	161 (100)	82 (100)	79 (100)	1
Flavored e-liquid	161 (100)	82 (100)	79 (100)	1
Non-nicotine e-liquid	18 (11.2)	11 (13.4)	7 (8.9)	0.359
Nicotine-containing e-liquid	161 (100)	82 (100)	79 (100)	1
E-cigarette derivative	6 (3.7)	2 (2.4)	4 (5.1)	0.437
**Promotion**	161 (100)	82 (100)	79 (100)	1
Marketing materials inside the store	161 (100)	82 (100)	79 (100)	1
Marketing materials outside the store	52 (32.3)	25 (30.5)	27 (34.2)	0.512
Price shown	161 (100)	82 (100)	79 (100)	1
Flavors shown	161 (100)	82 (100)	79 (100)	1
Discounts	55 (34.2)	26 (31.7)	29 (36.7)	0.615
Free trial service	161 (100)	82 (100)	79 (100)	1
Vaping in-‐store (customers and/or staff)	28 (17.4)	15 (18.3)	13 (16.5)	0.810
Slogans	93 (57.8)	46 (56.1)	47 (59.5)	0.663
**Type of QR code within the store**	88 (54.7)	50 (61.0)	38 (48.1)	0.010
QR code of member	47 (29.2)	29 (29.4)	18 (17.6)	0.434
WeChat QR code of a store contact	41 (25.5)	29 (35.4)	12 (15.2)	0.003
QR code of ‘Shansong’ (express delivery)	28 (17.4)	25 (30.5)	3 (3.8)	<0.001
QR code of brand WeChat official account	18 (11.2)	5 (6.1)	13 (16.5)	0.037

### Promotion

In each e-cigarette specialty store, the prices of e-cigarette products and the e-liquid flavor were clearly labelled (Table 2). Fifty-five retailers (34.2%) offered promotions such as special pricing or multipack discounts. Each e-cigarette specialty store provided e-liquid samples and disposable plastic covers to encourage people to take free trials of their e-liquids with a variety of flavors. During our observation, employees or customers were vaping in 31 of the stores (19.7%). Ninety-three stores (57.8%) prominently displayed brand slogans, such as ‘Take a RELX, Relax Your Moment’. Of the 161 specialty stores, 32.3% had e-cigarette marketing materials outside the stores, and 100% had e-cigarette marketing materials inside the stores. There were no significant differences in the types of promotions between the two cities.

Eighty-eight specialty stores (54.7%) displayed different QR codes within their premises. Forty-seven stores showcased membership QR codes, through which people could become members of brands and enjoy membership benefits. Forty-one stores displayed their store-specific QR codes, which typically represent the store owner’s WeChat ID. Customers can scan this QR code to befriend the store contact on WeChat, from which customers can obtain new product information and even order products online. Twenty-eight stores displayed a ‘Shansong’ QR code, enabling customers to conveniently place orders for a fast e-cigarette delivery experience. Additionally, 18 stores exhibited official QR codes for their brands’ WeChat public accounts, which serve as hubs for product information, customer support, promotions, community interaction, and industry updates. E-cigarette stores in Shanghai feature a wider variety of QR code types compared to those in Chengdu (p=0.01).

### Marketing materials

Each specialty store had some form of marketing material for e-cigarettes (Table 3). A distinctive characteristic of single-brand marketing materials was the high degree of duplication in different stores of the same brand. In total, 149 stores (92.5%) exhibited illuminated banners, focusing on themes such as product introduction (n=125; 78.6%), brand identity (n=110; 69.2%), promotional activities (n=8; 5.0%), and youth protection (n=9; 5.7%). Over 70% of the stores (n=116) showcased posters with themes of product introduction (n=50; 43.1%), promotional activities (n=36; 31.0%), youth protection (n=29; 25.0%), and brand identity (n=23; 19.8%). A few specialty stores played e-cigarette videos (n=10; 6.2%), sourced from particular e-cigarette manufacturers, featuring details about the products (n=7; 70.0%), brand stories (n=4; 40.0%), and youth protection (n=2; 20.0%). There were no significant differences in the types of marketing materials between the two cities.

**Table 3:** Marketing materials in e-cigarette specialty stores (N=161)

*Marketing materials*	*Total n (%)*	*Shanghai n (%)*	*Chengdu n (%)*	*p*
**Category of different types**	161 (100)	82 (100)	79 (100)	1
Table tent	155 (96.3)	82 (100)	73 (92.4)	0.030
Illuminated banner	149 (92.5)	78 (91.5)	74 (94.7)	0.950
Poster	116 (72.0)	59 (72.0)	57 (72.2)	1
Brochure	33 (20.5)	17 (20.7)	16 (20.3)	1
Videos	10 (6.2)	5 (6.1)	5 (6.3)	1
**Number of characters**				**<0.001**
0	54 (33.5)	18 (22.0)	36 (45.6)	
1	69 (42.9)	33 (40.2)	36 (45.6)	
2	24 (14.9)	17 (20.7)	7 (8.9)	
≥3	14 (8.6)	14 (17.1)	0 (0)	
**Character gender**				**0.005**
No character	54 (33.5)	18 (22.0)	36 (45.6)	
Male	38 (23.6)	22 (26.8)	16 (20.3)	
Female	35 (21.7)	18 (22.0)	17 (21.5)	
Both	34 (21.1)	24 (29.3)	10 (12.7)	
**Age** (years)				**0.009**
No character	54 (33.5)	18 (22.0)	36 (45.6)	
Youth	104 (64.6)	63 (76.8)	41 (51.9)	
Middle-aged	1 (0.6)	1 (1.2)	0 (0)	
The elder	1 (0.6)	0 (0)	1 (1.3)	
Mixed age	1 (0.6)	0 (0)	1 (1.3)	
**Whether the character is vaping**				**0.005**
No character	54 (33.5)	18 (22.0)	36 (45.6)	
Vaping	8 (5.0)	5 (6.1)	3 (3.8)	
Holding, not vaping	97 (60.2)	59 (72.0)	38 (48.1)	
Not touching	2 (1.2)	0 (0)	2 (2.5)	

Images of characters (66.5%) were a common feature of the marketing materials of e-cigarette specialty stores, with an almost equal representation of males and females. Youth images (64.6%) were frequently used on marketing materials. Among the individuals included in the marketing materials, 7.5% were vaping. There were differences between the two cities in terms of the character features in marketing materials.

### Age restriction and health warnings

The 161 specialty stores did not impose any signage about age restrictions for minors’ entry (Table 4). Signage prohibiting both purchase and use by minors was displayed in 87.6% (n=141) of the stores. Seventy-two stores (44.7%) had signage about requests to show a Chinese residency ID card at time of purchase. Only a relatively small number of specialty stores (n=51; 31.7%) displayed health warnings, with 23% of the stores stating that smoking is harmful to health and 19.9% of the stores citing the addictive nature of nicotine. There were no significant differences in the overall age restrictions and health warnings between the shops in the two cities.

**Table 4:** Age restriction and health warning signage in e-cigarette specialty stores (N=161)

*Signage*	*Total n (%)*	*Shanghai n (%)*	*Chengdu n (%)*	*p*
**Age restriction**	142 (88.2)	75 (91.5)	67 (84.8)	0.287
Prohibit the purchase and use of e-cigarettes by minors	141 (87.6)	74 (90.2)	67 (84.8)	0.296
Show your Chinese residency ID card for purchase	72 (44.7)	34 (41.5)	55 (69.6)	<0.001
Prohibit entry of minors	0 (0)	0 (0)	0 (0)	1
**Health warning**	51 (31.7)	22 (26.8)	29 (36.7)	0.239
Smoking is harmful to health	37 (23.0)	19 (23.2)	18 (22.8)	0.954
Nicotine is addictive	32 (19.9)	8 (9.8)	24 (30.4)	0.001

## DISCUSSION

This study helps to advance our scientific knowledge of the characteristics of these increasingly popular retail environments. Our study identified recreational and stylish store decorations, highly visible product displays, diverse product ranges, ample marketing materials, complimentary trial services, the absence of restrictions on minors’ entry, and a lack of health warnings in these specialty stores. All these features collectively contribute to the significant role played by specialty stores in exposing youth to e-cigarette products and advertising, and the normalization of e-cigarette use.

### Shop characteristics

Our research indicates that the majority of these specialty stores adhere to a single-brand model. These single-brand outlets function as crucial contact points, facilitating a connection between brands and consumers and delivering a holistic brand experience. Single-brand stores allow e-cigarette manufacturers to have greater control over their brand image and customer experience compared to multi-brand specialty stores. Additionally, a single-brand store can focus its marketing efforts more effectively, creating a unique brand identity that attracts its target customers. A notable feature of single-brand retailers is their uniform and modern decoration across all branches of the same brand, maintaining a consistent design style that supports instant brand recognition.

Many of these stores are compact (<10 m^2^) which is both space- and cost-efficient. Moreover, their size naturally fosters a closer rapport between store personnel and patrons, which is convenient for providing tailored recommendations and guidance. Geographically, predominant stores are situated at underground (37.8%) or at ground level (46.0%). Stores on the ground floor offer visibility and easy accessibility for pedestrians, while those underground often appeal to commuters, especially near subways.

### Product displays

With the rise of youth e-cigarette use, it is worth considering restrictions on youth exposure to e-cigarette marketing and access to these products. In each specialty shop we observed, e-cigarette displays were highly visible, exposing all passersby to marketing. This observation aligns with findings from studies conducted in the United States^22^, Canada^17^, the United Kingdom^23^, New Zealand^19^, and Australia^20^. A study found that greater visibility of e-cigarette retail displays reduced the perceived harm of smoking^24^. Hence, such widespread and high visibility of product displays among retailers indicates an urgent need for restrictions on product displays.

### Promotions

More so than in other studies^8,14,16^, all the specialty shops we observed offered free e-liquid samples, encouraging customers to experiment with various nicotine and e-liquid flavor options. During our observation period, thirty-one stores (19.7%) had staff or customers vaping in-store, lower than in previous studies in Australia^20^. This may be due to our observation time, as the flow of customers in business areas drops sharply on weekdays.

Vaping in specialty stores is concerning. First, the harmful substances released into the air by vaping in stores can be harmful to people in the surrounding area. Second, the e-cigarette specialty stores we observed were located in business districts that were frequently visited by youth. Observing vaping behaviors in public places may contribute to positive attitudes towards e-cigarette use and lead to further vaping initiation among the youth. Third, vaping in these stores may normalize e-cigarette use and promote social acceptance of e-cigarette products, posing a threat to the overall smoke-free environment.

Our research found that some individuals may have used e-cigarettes inside stores. This could be because, at the time of our observation, Chengdu and Shanghai had not included e-cigarettes in smoke-free regulations. The omission of e-cigarettes in the majority of subnational smoke-free laws may lead to confusion and poor adherence to existing smoke-free regulations^25^, impeding efforts to achieve a 100% smoke-free environment. Hence, cities that have enacted tobacco control regulations should involve e-cigarette use in their smoke-free regulations.

Although China has banned the online sale and advertising of e-cigarettes, e-cigarette specialty stores have not abandoned online marketing and sales. Our study revealed that nearly 20% of specialty stores displayed ‘Shansong’ QR codes and 26% featured shop WeChat QR codes, which made it possible to buy e-cigarettes without visiting the store. This covert form of online sale is challenging to regulate. Furthermore, e-cigarette specialty stores feature QR codes about membership and the official WeChat accounts of their brands. Scanning these codes provides users with extensive product and brand information, constituting a form of online marketing. Previous studies have revealed detailed contact information, including social media accounts and offline store locations, on the official websites of Chinese e-cigarette manufacturers^26^, highlighting the integrated online and offline marketing strategies of these companies. Thus, merely prohibiting the online sale and advertising of e-cigarettes may not be sufficient. Clear definitions of online sales and advertising, combined with rigorous enforcement of these definitions, can contribute to reducing covert online sales and marketing activities.

### Marketing materials

A meta-analysis of prospective studies found that exposure to e-cigarette advertisements, specifically in retail stores, increased e-cigarette use among adolescents and young adults^27^. Due to the online sales and advertising ban since 2019^5^, retailers have become the main access point for e-cigarette marketing. Our study observed that all specialty stores had e-cigarette advertising, higher than those reported by other countries^8,28^. E-cigarettes were widely promoted at specialty stores with diverse kinds of marketing materials, including posters, table tents, illuminated banners, and brochures. Unlike international vape shops which independently develop marketing strategies^29^, specialty stores in China rely heavily on e-cigarette manufacturers. This is because marketing and merchandising materials, such as signage and price promotions, are uniformly designed and printed by manufacturers. This uniform approach enhances the brand’s overall impression and appeal.

E-cigarette marketing materials typically incorporate elements such as a brand logo, images of characters, and some description of the product^30^. Eye-tracking studies have revealed that participants focus most on characters, followed by product descriptors and finally brand logos^30^. Our study found that 66.5% of the retailers displayed images of models on their marketing materials, consistent with previous studies^31^. This strategy likely aims to make their marketing message more readily accepted among the youth^30^. Notably, on these marketing materials with figures, 7.5% of the characters were using e-cigarettes. A previous experiment in the US found that advertisements showing a person using an e-cigarette created more interest than advertisements not showing an e-cigarette^32^.

### Age restriction and minor protection

E-cigarette marketing, similar to other tobacco product marketing, is unlikely to directly target minors^33^. Our study did not find any direct marketing of e-cigarettes to adolescents. Additionally, some marketing materials even contained information about adolescent protection, and this finding is consistent with earlier studies on the official websites of Chinese e-cigarette manufacturers^26^. However, this does not mean that teenagers are never exposed to e-cigarette retailers’ marketing. Our study revealed that none of the retailers displayed signage regarding age-related entry restrictions, a percentage that was significantly lower than what was reported in the US survey^8^. Furthermore, 12% of the stores had no age restriction for use and purchase. This lenient environment can inadvertently elevate teenagers’ exposure to e-cigarette marketing, which in turn increases their possibility of e-cigarette initiation^9^.

### Health warnings

Our study found that relatively few specialty stores displayed health warnings, a percentage that was lower than what was reported in the US survey^8^. Only 23% of the stores claimed that smoking is harmful to health, and 19.9% of the stores claimed that nicotine is addictive. Among those with a health warning, detailed information such as the risk of specific diseases was rarely observed. A lack of specific and detailed health warnings about these products may lead to underestimation of e-cigarette hazards. Experimental investigations have suggested that making e-cigarette warning labels more prominent and noticeable can enhance young adults’ attention to warnings and recall of health messages and affect product perceptions^34^. Hence, a specific and prominent health warning should be applied in retailers’ marketing materials.

### Policy implications

The Administrative Measures of E-cigarettes^35^, which were released in 2022, have established clear regulations addressing some key issues highlighted in our study. First, prohibit e-cigarette stores from exclusively selling single-brand e-cigarette products, which may reduce the appeal and recognition of e-cigarette brands. Second, e-cigarette advertising is regulated under existing laws, regulations, and rules applicable to tobacco advertising, which have rectified the previously unregulated situation of offline e-cigarette advertising. According to the National Advertisement Law Amendment^10^, tobacco advertisements are prohibited in the mass media, public places, public transportation and outdoors. Nonetheless, the Advertising Law lacks a precise definition of public places, leading to ongoing debates about whether tobacco stores should be classified as such and whether advertising should be banned within these establishments. The absence of a specific definition and detailed enforcement measures of e-cigarette advertising in retailer environments allows some retailers to act as if no regulations apply to their stores. Hence, this issue has not been properly solved. Third, e-cigarette operators are required to display signs in prominent locations stating that sale of e-cigarettes to minors is prohibited and should request identification from individuals who are difficult to identify as adults. Finally, the sale of non-tobacco flavored e-cigarettes is forbidden, which may reduce the appeal of e-cigarettes.

While the Administrative Measures of E-Cigarettes has partially addressed some issues identified in our research, certain findings from our study still emphasize the need to strengthen regulations. First, e-cigarettes should be incorporated into smoke-free legislation. As of December 2023, 24 cities have enacted local smoke-free legislation, with 9 of these cities including e-cigarettes within their smoke-free laws^36^. Therefore, there is a need to advocate for the inclusion of e-cigarettes in smoke-free legislation in the future. Second, a more detailed ban on tobacco advertising, promotion, and sponsorship is essential for effective supervision of e-cigarettes. The definition of tobacco advertising, promotion, and sponsorship needs to be clarified to provide law enforcement agencies with clear guidelines for enforcement. Additionally, regulations should explicitly state that product display constitutes a form of tobacco advertising. Policymakers and regulatory agencies should take steps to minimize youth exposure to e-cigarette products and marketing in the retail environment by restricting product display and marketing.

### Strengths and limitations

Our study has two key strengths. First, to our knowledge, this is the first study to empirically assess the in-store marketing of e-cigarettes in China. This highlights the growing marketing of e-cigarettes and the need to monitor e-cigarette marketing practices. Second, our study was conducted in 2021, before the implementation of the E-cigarette Management Measures, documenting the overall situation of China’s e-cigarette specialty stores during the most lenient period of regulation. This provides baseline data for comparison with future surveillance efforts to document the impact of the implementation of Administrative Measures of E-cigarettes and subsequent research on vape shop marketing practices.

The limitations of the study can be attributed to the nature of our sampling. First, our study was geographically restricted to Shanghai and Chengdu, limiting its generalizability to other cities and rural areas. Second, only specialty stores were included, excluding other retail environments like convenience stores and supermarkets. Third, the stores we observed were located in popular business districts, which may introduce selection bias as these stores might differ in brand, marketing strategies, and customer demographics compared to those in other areas. Hence, a larger study across multiple regions and retail environments is needed to precisely estimate e-cigarettes marketing practice in China.

## CONCLUSIONS

Our study revealed that e-cigarette specialty stores exhibited highly visible product displays, varied product selections, and free trial services. Additionally, these stores featured abundant marketing materials, absent restrictions on entry for minors, and a lack of health warnings. These findings support stricter regulations on product displays, marketing practices, age restrictions and health warnings within the retail environment. Our study also highlighted the need for continued surveillance to comprehend how these marketing practices influence individuals’ exposure to and use of e-cigarettes.

## Supplementary Material



## Data Availability

The data supporting this research are available from the authors on reasonable request.
