# Supplementary material for: An observational study of the marketing practice of e-cigarette specialty stores in two large cities in China: Is there potential to normalize the use of e-cigarettes?
Source: Tob Induc Dis. 2024 Sep 14;22:10.18332/tid/191840. doi: 10.18332/tid/191840 (PMC11401606; doi:10.18332/tid/191840)
Supplement: Supplementary file 1 [file TID-22-160-s1.pdf]

Table S1 Checklist for e-cigarette specialty store

Section	Item	Definition
**Basic information**	Specialty store classification	Single-brand (exclusively sell products from a specific e-cigarette brand) or multibrand (sell a variety of e-cigarette products from different brands)
	Brand name	Use of texts or pictures to display brand name (e.g., RELX)
	Store area	Visually estimated total space utilized for the display and sale of e-cigarette products
	Location	Detailed information about the location and floor of the e-cigarette store
**Product display**	E-cigarette types	Disposable (single-use devices with prefilled e-liquid and be discarded after the e-liquid is depleted or the battery dies) or rechargeable kits (reusable devices consist of a rechargeable battery and a refillable tank or pod)
	Number of e-liquid flavors	Total count of distinct e-liquids taste (such as fruits, desserts, beverages and menthol) available in the store
	Zero-nicotine e-liquid	Type of e-liquid contains flavorings and other ingredients but is free of nicotine
	E-cigarette derivative	Supplementary items enhance the use and functionality of e-cigarette, which include, but are not limited to, items like carrying cases, and customization elements like skins or wraps

Section	Item	Definition
**Promotion**	Discounts	Reduction in the regular price of a product or service (e.g., buy two get one free)
	Marketing materials	A variety of communication tools and media specifically designed to promote and inform target audiences about e-cigarette products and related brands (e.g., brochures, posters, and videos)
	QR codes within the stores	Specific QR codes displayed by the store to facilitate communication and interaction with customers (e.g., member QR code)
**Age restrictions**	Age verification for store entry	Use of texts or pictures to inform about the age restriction for entry (e.g., verify age before permitting entry to the store)
	Age-restricted statement for e-cigarette usage	Use of texts or pictures to inform about the age restriction for e-cigarette use (e.g., minors are prohibited from using e-cigarettes)
	Age-restricted statement for e-cigarette purchase	Use of texts, pictures or videos to inform about the minimum age requirement for e-cigarette purchase (e.g., minors are not allowed to purchase e-cigarettes)
**Health warning**	Statement of health warning	Notices or labels displayed within the store to inform consumers about health risks associated with the use of e-cigarettes (e.g., product contains nicotine, which is addictive) through texts, pictures or videos

Table S2. Location distribution of e-cigarette specialty store (N=161)

City	District	Business area	Frequency (n, %)
Shanghai	Pudong New District	Century Avenue	13 (8.1)
	Huangpu District	Tianzi Fang / Dapuqiao	18 (11.2)
	Huangpu District	Nanjing Road	11 (6.8)
	Yangpu District	Wujiao Chang	8 (5.0)
	Xuhui District	Xujia Hui	10 (6.2)
	Jing'an District	Jingan Temple	5 (3.1)
	Changning District	Zhongshan Park	7 (4.3)
	Minhang District	Qibao Ancient Town	10 (6.2)
Chengdu	Jinjiang District	Chunxi Road	45 (28.0)
	Jinjiang District	Ciqi Kou	12 (7.5)
	Chenghua District	University of Electronic Science and Technology University of China/ Jianshe Road	10 (6.2)
	Qingyang District	Tianfu Square	7 (4.3)
	Wuhou District	Wuhou Temple	5 (3.2)

© 2024 Deng H. et al.
